# Cu-Ag/SBA-15 nano catalysts for the control of microorganisms in water

**DOI:** 10.1186/s11671-024-04176-5

**Published:** 2025-01-27

**Authors:** Saidulu Ganji, Ramesh Kola, Kumaraswamy Gullapelli, Ramesh Martha

**Affiliations:** 1 Department of Chemistry, Chaitanya Bharathi Institute of Technology(A), Hyderabad, 500075 India; 2 Department of Physics and Chemistry, Mahatma Gandhi Institute of Technology(A), Hyderabad, 500075 India

**Keywords:** Cu-Ag/SBA-15 nano alloy catalysts, Microorganism control, High surface area

## Abstract

Because of their uniform and regular channels, adjustable pore size, large surface area, controllable wall composition, high hydrothermal stability, ease of functional modification, and good accessibility of larger reactant molecules, mesoporous siliceous SBA-15 is of excellent catalyst carrier that is highly versatile and has been used extensively to prepare a variety of supported catalysts with ideal catalytic properties. In this study, we report the synthesis, characterization, and catalytic application of Cu-Ag/ SBA-15 nanoalloy catalysts towards the control of microorganisms in drinking water has been reported. The Cu-Ag/SBA-15 nanoalloy catalysts with different molar mass ratio of copper to silver (Cu:Ag = 1: 0, 0.75: 0.25, 0.5: 0.5, 0.25: 0.75, 0: 1) keeping 1weight % total loading of copper and silver metals on SBA-15 support have been prepared by incipient wetness impregnation method and characterized by various characterization techniques like, low angle XRD, wide angle XRD, N_2_-physcisorption and scanning electron microscopy techniques. The anti-bacterial activity of the catalysts was measured qualitatively by testing the presence of coliforms in water after contacting with the catalyst at room temperature. These nanoalloy catalysts found to be effective in controlling the microorganisms in drinking water. Among the series of the catalysts prepared, 0.25Cu-0.75Ag /SBA-15 catalyst showed superior catalytic activity. The high catalytic performance of the catalyst is due to its high surface area.

## Introduction

Worldwide, there is now a severe crisis due to the growing need for clean water and the depletion of available supplies brought on by the growth of industries, population growth, and protracted droughts. It leads to a need for the creation of novel, workable, and financially appealing technologies that permit sensible water use. Millions of people die each year from illnesses brought on by contaminated water, with over 4 billion people thought to have extremely restricted access to clean water [1]. It is reasonable to anticipate that such figures will rise in the future in line with the rising levels of environmental contamination brought on by the deposition of dangerous compounds into the natural water cycle [2]. The development of low-cost, high-performing water and wastewater treatment technologies is necessary due to the low quality of natural water and clean water shortage [3, 4].

When used as standalone or combined procedures, traditional water treatment techniques (such as filtration, activated carbon adsorption, flocculation/sedimentation, coagulation/flocculation/ sedimentation, and chlorination) are unable to completely remove microorganisms and hazardous inorganic and organic compounds from drinking water sources. As a result, new methods of treating wastewater and water, such as membrane technology, UV disinfection, and "Advanced Oxidation Processes," are being developed [5, 6]. Nevertheless, those techniques do not result in the complete neutralization or elimination of contaminants—rather, they just allow for the concentration or transfer of impurities to another phase. Although chlorination is typically used in disinfection processes, it can also produce byproducts that are carcinogenic and mutagenic [6]. Reverse osmosis, ultrafiltration, nanofiltration, and microfiltration are examples of chemical and membrane processes that have significant operating costs and have the potential to produce hazardous secondary pollutants that enter ecosystems. Membrane technologies are severely constrained, even though they have emerged as a viable substitute for traditional treatment techniques [7]. First off, those procedures only guarantee the physical separation of organic and inorganic components, as well as microbes. Because of this, the concentrated stream—roughly 10% of the treated water volume—contains pathogenic microorganisms that are active and can seriously harm the feed stream or deposition site [7].

For millennia, people have been aware of metallic silver's antimicrobial properties [8]. The dissolution of silver ions from the bulk silver's surface is the source of this advantageous characteristic. Unlike when using silver ions, bulk silver has a stable and long-lasting biocidal impact. However, due to its high cost and slow ion release rate, bulk silver is challenging to employ in household or industrial settings. A new method for applying silver antibacterial agents has emerged recently with the use of silver nanoparticles due to their large surface to volume ratio. Numerous studies have been conducted to determine the bactericidal potential of nanoparticles and their potential uses in the paint, textile, health, and plastics industries [9–14]. However, in view of green chemistry requirements, it is necessary to use reusable heterogeneous catalysts in the disinfection water. It has been reported that the Ag based catalysts have been effectively controls the microorganisms in drinking water [15–18]. Mesoporous materials have attracted a lot of attention due to their potential use in absorption, separation, catalysis, and other processes. SBA-15 is among the best catalytic supports in the mesoporous material family due to its large and constant pore size, high porosity, and large surface area. These features provide a dependable and well-isolated environment for the creation of nanoparticles. [19–21] In the present study, we describe the preparation of Cu-Ag/SBA-15 nanoalloy catalysts and its catalytic activity in control of microorganisms in drinking water.

## Experimental

### Preparation of mesoporous SBA-15

Mesoporous silica SBA-15 was prepared accordance with previous methods. [19–25] In general, SBA-15 was prepared by hydrothermal method in acidic medium using P_123_ polymer as structure directing agent and TEOS as silica source. A solution of EO_20_PO_70_EO_20_: 2 M HCl: TEOS: H_2_O = 2: 60: 4.25: 15 (mass ratio) was prepared, stirred for 12 h at 40 °C and then hydrothermally treated at 100 °C under static conditions for 12 h, subsequently filtered, dried at 100 °C and calcined at 550 °C for 8 h, the yielded white powder is mesoporous silica SBA-15.

### Preparation of Cu-Ag/SBA-15 catalysts

The Cu-Ag/SBA-15 catalysts have been prepared by incipient wetness impregnation method by taking the requisite amount of homogeneous mixed salt solutions of Cu and Ag with high dilution with the composition of copper and silver in the finished catalysts with the molar mass ratio of copper to silver (Cu:Ag = 1: 0, 0.75: 0.25, 0.5: 0.5, 0.25: 0.75, 0: 1) keeping 1weight % total loading of copper and silver metals on SBA-15 support. In a typical procedure, 1 g the 0.5Cu-0.5Ag/ SBA-15 was prepared by taking 0.99grs of SBA-15, aqueous solution containing 0.0787gr of AgNO_3_ and 0.19gr of Cu(NO_3_)_2_.3H_2_O. The mixture was evaporated to dryness and the catalyst samples were dried at 100 °C for 12 h and calcined at 450 °C for 6 h. The catalysts are labelled as (a) SBA-15, (b) 1%Cu/SBA-15, (c) 1%Ag/SBA-15, (d) 0.25Cu-0.75Ag/SBA-15, (e) 0.5Cu-0.5Ag/SBA-15 (f) 0.75Cu-0.25Ag/SBA-15. The prepared Cu-Ag/ SBA-15 nanoalloy materials were characterized by low angle XRD, wide-angle XRD, nitrogen adsorption–desorption and SEM techniques.

### Characterization of Cu-Ag/SBA-15 catalysts

The X-ray diffraction (XRD) patterns were recorded at room temperature using a Rigaku, Multiflex, diffractometer with a nickel filtered CuKα radiation of wave length 1.5418 A° at a power of 40 kW and a current of 100 mA in the 2 h range of 0.5–5° and 5–80° for the verification of SBA-15 structural ordering and Cu, Ag phase behavior respectively.

N_2_ adsorption–desorption isotherms were recorded for catalysts using a Tristar 3000 V 6.08A instrument (M/S Micromeritics Instruments Corporation, USA) at -196 °C. The samples were outgassed at 200 °C for 4 h before the measurement. BET method was used to calculate the surface areas using the amount of N_2_ adsorbed at -196 °C. The scanning electron microscopy images were recorded using a Hitachi S-520 SEM instrument. ICP-MS studies were made a simultaneous ICP-AES allied analytical system (Perkin Elmer 3100XL).

### Catalytic activity test

Using 0.1 g of catalyst, the activities of each catalyst were examined for control microorganisms in raw water. In a 100 ml sterile transparent tube with a screw cap, 0.1 g of the catalyst was further dipped in 50 cm^3^ of raw water (pond water, which is rich in microorganisms with E. coli) and agitated for 1 h in batch mode at room temperature. The catalyst was removed after an hour, and the water's bacterial content was quantitatively examined.

### Quantitative analysis of microorganisms

Raw water to be analyzed was taken and serially diluted to 10^−6^ dilution in a series of six test tubes. One millilitre of the raw water was taken in the first test tube containing 9 ml of saline solution and from it 1 ml was taken into the second test tube also containing 9 ml of saline solution. This process is repeated to the last, i.e., 10^−6^ dilution. Since the raw water contains large number of microorganisms, which cannot be counted, it is necessary to dilute it. From each test tube 0.1 ml of water was taken and was spread using a spreader, on a Petri-plate containing solidified nutrient agar and were incubated at 310 K for 24 h [17]. This entire process was done in the UV laminar airflow. The number of colonies grown were then counted after incubation.

## Results and discussion

### Characterization of Cu-Ag/SBA-15 nanoalloy catalysts by low-angle XRD

**Fig. 1:**
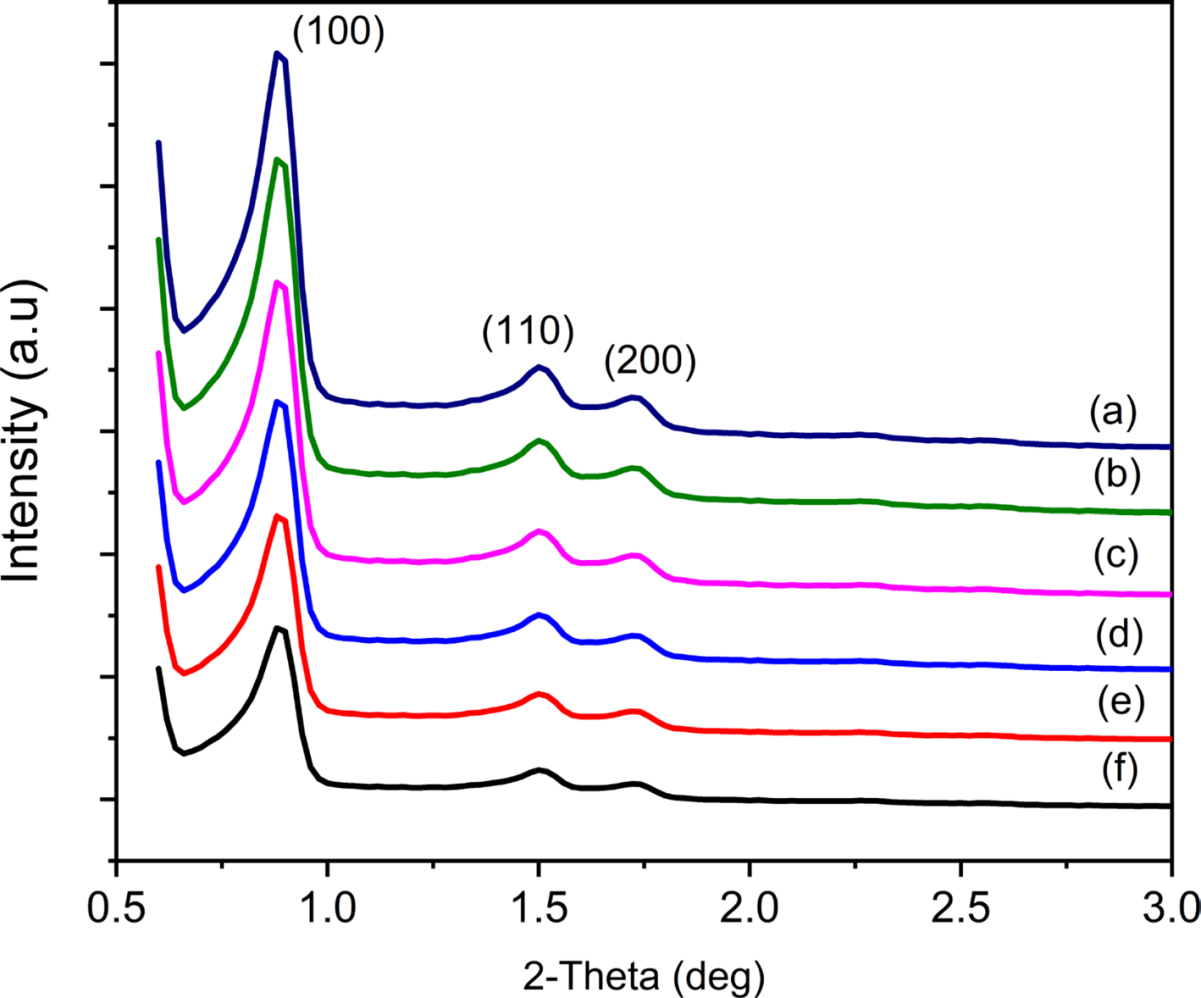
Low-angle XRD patterns of Cu-Ag/SBA-15 nanoalloy materials. **a** SBA-15, **b** 1%Cu/SBA-15, **c** 1%Ag/SBA-15, **d** 0.25Cu-0.75Ag/SBA-15, **e** 0.5Cu-0.5Ag /SBA-15, **f** 0.75Cu-0.25Ag/SBA-15

To assess the structural integrity of the catalysts, low-angle XRD patterns of Cu-Ag/SBA-15 catalysts including SBA-15 were recorded and the resultant patterns are shown in Fig. 1. The XRD patterns of Cu-Ag/SBA-15 nanoalloy show three well-resolved diffraction peaks at about (2θ = 0.89, 1.50, and 1.73º) that are assigned as (100), (110) and (200) reflections, associated with p6mm morphology. These XRD results confirm that hexagonal SBA-15 with a large lattice parameter is thermally stable and mesoscopically well-ordered. In other words, the structure of siliceous SBA-15 has been retained even after loading of Cu and Ag. The unit cell parameter and other structural parameters determined for Cu-Ag/SBA-15 were shown in Table 1.

**Table 1:** Physico-chemical characteristics of the catalysts

Catalyst	S_BET_^a^ m^2^/g	V_t_^b^ cc/g	D^c^ nm	d_1oo_^d^ nm	a_o_^e^ nm	t^f^ nm
SBA-15	688	1.08	6.3	9.65	11.1	4.8
1%Cu/SBA-15	560	0.84	6	9.45	10.9	4.9
1%Ag/SBA-15	612	0.94	6.1	9.09	10.8	4.7
0.25Cu-0.75Ag /SBA-15	652	1.01	6.2	9.41	10.9	4.7
0.5Cu-0.5Ag/SBA-15	632	0.93	6.1	9.45	10.9	4.8
0.75Cu-0.25Ag/SBA-15	620	0.91	6	9.44	10.9	4.9

^a^BET surface area, ^b^total pore volume, ^c^BJH pore diameter, ^d^periodicity of SBA-15 derived from low angle XRD, ^e^unit cell length (a_0_ = 2d_100_/√3), ^f^pore wall thickness (t = a_0_−D)

### Characterization of Cu-Ag/SBA-15 nanoalloy catalysts by wide-angle XRD

**Fig. 2:**
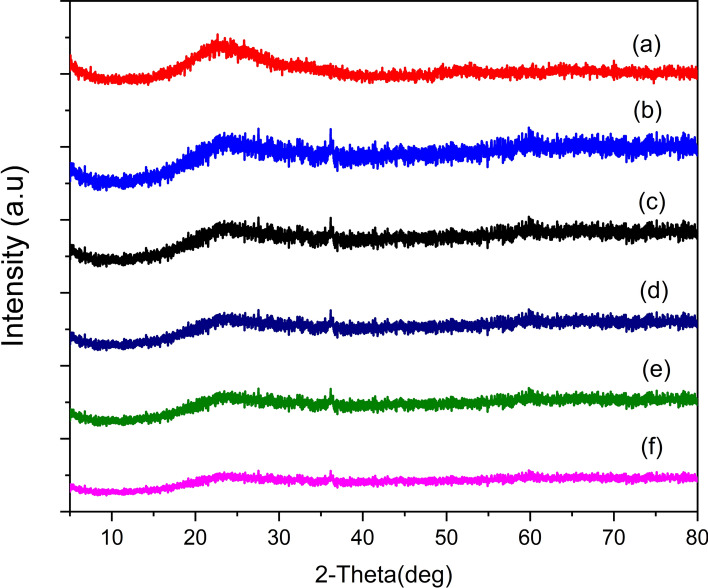
Wide-angle XRD patterns of Cu-Ag/SBA-15 nanoalloy materials. **a** SBA-15, **b** 1%Cu/SBA-15, **c** 1%Ag/SBA-15, **d** 0.25Cu-0.75Ag/SBA-15, **e** 0.5Cu-0.5Ag /SBA-15, **f** 0.75Cu-0.25Ag/SBA-15

To assess the crystalline behavior of Cu and Ag species that are dispersed on the surface of SBA-15 support X-ray diffraction analysis was made in the 2θ range of 5–80 (Fig. 2). A large hump that was observed in all the sample at about 2θ range 20–30 corresponds to SiO_2_ in mesoporous silica. A small diffraction peak is observed in 1%Cu/SBA-15 catalyst at 2θ = 36.12° might be related to Cu(I). No corresponding metallic peaks of Cu or Ag were observed and a small diffraction peak at 2θ values at 36.27° was observed in all Cu-Ag/SBA-15, which might be indicates the formation of alloy formation.

### Characterization of Cu-Ag/SBA-15 nanoalloy catalysts by N_2_ adsorption–desorption isotherms

**Fig. 3:**
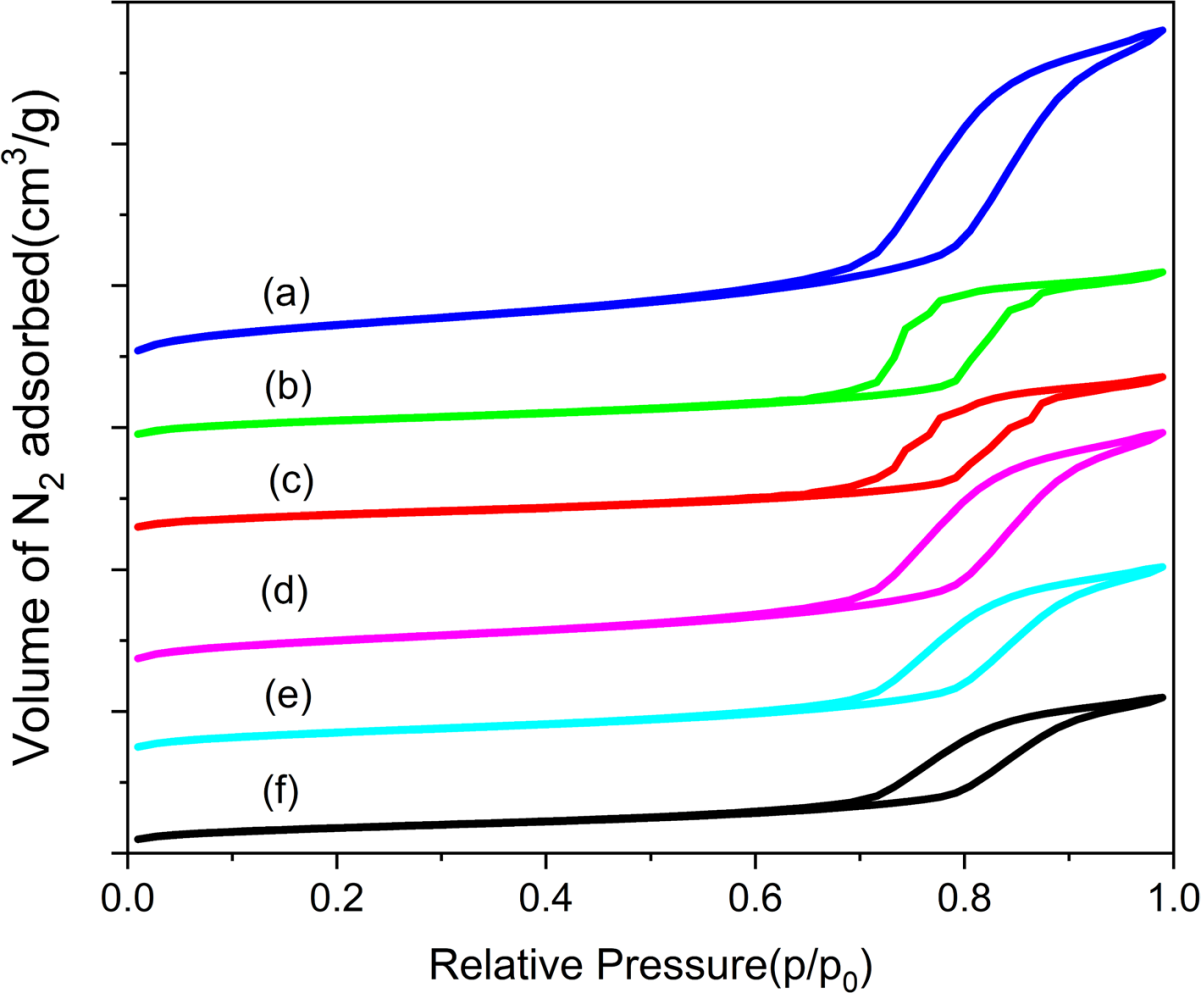
The nitrogen adsorption–desorption isotherms of Cu-Ag/SBA-15 nanoalloy materials. **a** SBA-15, **b** 1%Cu/SBA-15, **c** 1%Ag/SBA-15, **d** 0.25Cu-0.75Ag/SBA-15, **e** 0.5Cu-0.5Ag /SBA-15, **f** 0.75Cu-0.25Ag/SBA-15

Figure 3 shows the nitrogen adsorption–desorption isotherms for parent SBA-15 and Cu-Ag nanoalloy materials. The isotherms obtained for parent SBA-15 and Cu-Ag nanoalloy materials are of type IV and exhibited a hysteresis loop of H1 type in accordance with the IUPAC classification [19, 20], which indicates that the parent SBA-15 mesoporous structure was well maintained during the stages of catalyst preparation. The isotherms feature with sharp adsorption and desorption branches indicating a narrow meso pore size distribution (Fig. 3). The physico-chemical characteristics of the catalysts have been included in Table 1. It is observed that the decrease in surface area and pore volume of the catalysts after the loading of Cu and Ag on to the SBA-15. The decrease in the surface area and pore volume of the catalysts is due to the pore blockage by the metal nanoparticles.

### Characterization of samples by scanning electron microscopy

**Fig. 4:**
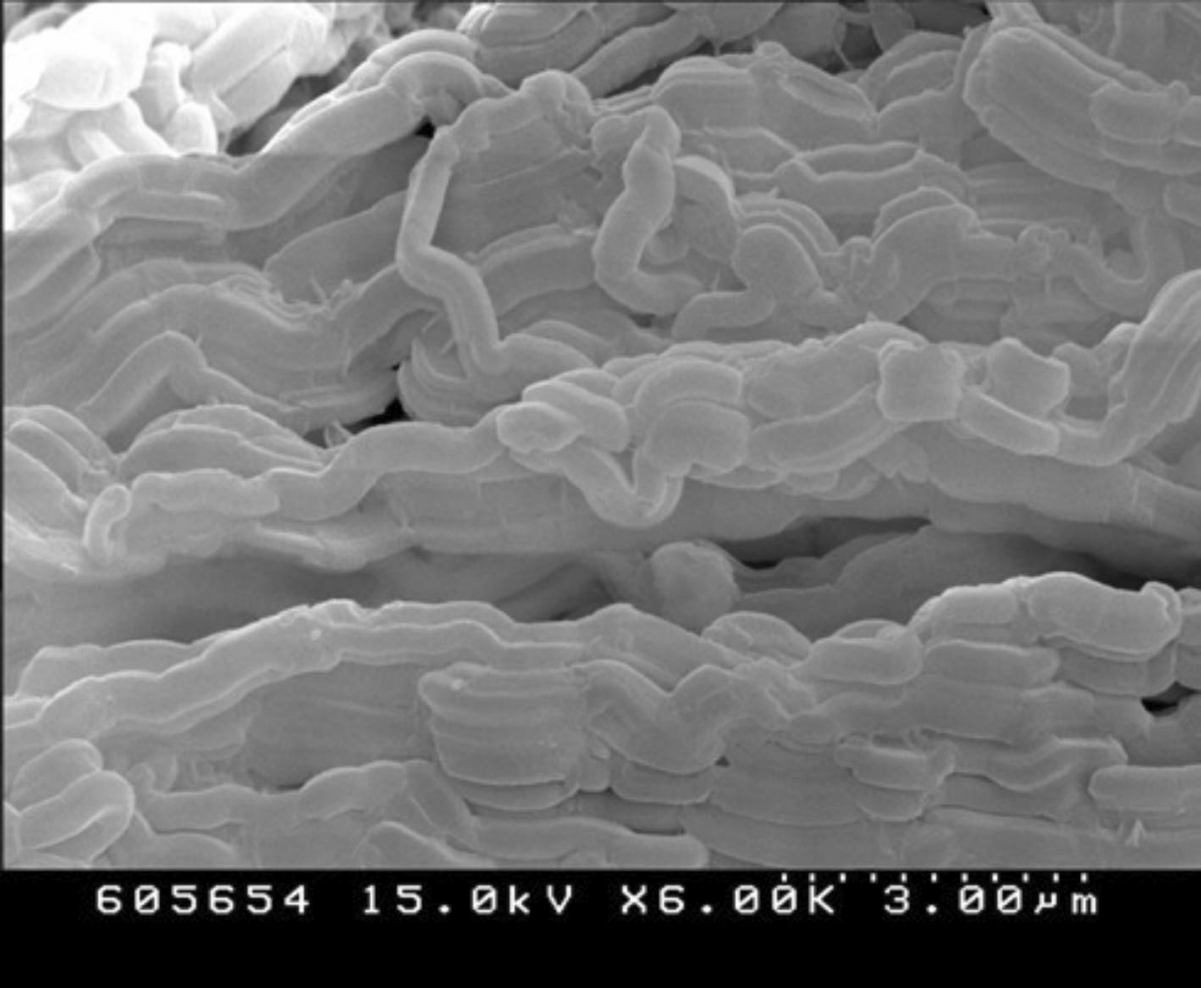
Scanning Electron Microscopy image of 0.25Cu-0.75Ag/SBA-15

Scanning electron microscopic image of 0.25Cu-0.75Ag/SBA-15 has been displayed in Fig. 4. The wormhole-like morphology of parent SBA-15 is well preserved in the 0.25Cu-0.75Ag /SBA-15 nanoalloy material.

### Destruction of microorganisms present in drinking water

To a 50 ml of water containing coliforms, 0.5 g of 1%Cu/SBA-15 catalyst was added and stirred for 1 h, followed by separated the catalyst by a simple filtration. 1 ml of filtrate water that is to be analyzed is taken in a test tube containing 9 ml of saline water followed by a sequential dilution in a series of 6 test tubes each containing 9 ml of saline water. Similarly, 1 ml of raw water is taken in the first test tube containing 9 ml of saline solution and from it, 1 ml is taken into the second test tube also containg 9 ml of saline solution. This process is repeated 6 times from each test tube, 0.1 ml of the water is taken and is spread on the petri-plates using a spreader containing solidified nutrient agar and are incubated at 37 ºC for 24 h. The number of coliforms grown is then counted after incubation.

The microorganism destruction activity of Cu/SBA-15, Ag/SBA-15 and three Cu-Ag/SBA-15 catalysts was displayed in the following Tables. First, the destruction of microorganisms in water was checked with 0.5 g of 1%Cu/SBA-15 and 1%Ag/SBA-15 catalysts individually. From the results depicted in Tables 2 and 3, it is clear that the Ag/SBA- 15 catalyst is better in controlling the microorganisms compare with Cu/SBA-15.

**Table 2:** The microorganism destruction activity of Cu/SBA-15

Catalysts used	Bacterial count (CFU/ml) Samples at different dilutions					
	10^–1^	10^–2^	10^–3^	10^–4^	10^–5^	10^–6^
Raw water	9 × 10^8^	9.1 × 10^7^	8.9 × 10^6^	9.1 × 10^4^	9.1 × 10^3^	9.1 × 10^2^
Cu/SBA-15 (0.1 g)	7.2 × 10^6^	1.1 × 10^5^	2.6 × 10^3^	9.9 × 10^2^	88	Nil
Cu/SBA-15 (0.25 g)	3.6 × 10^4^	6.1 × 10^3^	88	39	Nil	Nil
Cu/SBA-15 (0.5 g)	1.2 × 10^3^	99	28	Nil	Nil	Nil

**Table 3:** The microorganism destruction activity of 1%Ag/SBA-15

Catalysts used	Bacterial count (CFU/ml) Samples at different dilutions					
	10^–1^	10^–2^	10^–3^	10^–4^	10^–5^	10^–6^
Raw water	9 × 10^8^	9.1 × 10^7^	8.9 × 10^6^	9.1 × 10^4^	9.1 × 10^3^	9.1 × 10^2^
Ag/SBA-15 (0.1 g)	4.7 × 10^4^	7.8 × 10^2^	319	119	Nil	Nil
Ag/SBA-15 (0.25 g)	3.6 × 10^4^	6.1 × 10^3^	88	39	Nil	Nil
Ag/SBA-15 (0.5 g)	7.4 × 10^2^	Nil	Nil	Nil	Nil	Nil

In the subsequent tests examined, the catalytic performance of several Cu-Ag/SBA-15 nanoalloy catalysts were depicted in Tables 3, 4 and 5. No coliforms were found after treating the water with the catalyst 0.25Cu-0.75Ag/SBA-15 at 0.25 g and 0.5 g level. Whereas with two other catalysts with the compositions (0.75Cu-0.25Ag/SBA-15 and 0.5Cu-0.5Ag/SBA-15), a few number of coliforms were detected. This superior activity of the 0.25Cu-0.75Cu/SBA-15 catalyst is due to its high surface area, i.e. 652m^2^/g. In the case of 0.5Cu-0.5Ag/SBA-15 and 0.75Cu-0.25Ag/SBA-15, the surface area is found 632m^2^/g and 620 m^2^/g respectively. It can be seen from the Table 1 that; the surface area of individual catalysts is lower than the surface area of alloy catalysts. Accordingly, the activity of the catalysts in suppressing the coliforms can be seen from Tables 4, 5 and 6.

**Table 4:** The microorganism destruction activity of Cu-Ag/SBA-15 at 0.1 g level

Catalysts used	Bacterial count (CFU/ml) Samples at different dilutions					
	10^–1^	10^–2^	10^–3^	10^–4^	10^–5^	10^–6^
Raw water	9 × 10^8^	9.1 × 10^7^	8.9 × 10^6^	9.1 × 10^4^	9.1 × 10^3^	9.1 × 10^2^
0.25Cu-0.75Ag/SBA-15	311	93	27	Nil	Nil	Nil
0.5Cu-0.5Ag/SBA-15	417	265	51	Nil	Nil	Nil
0.75Cu-0.25Ag/SBA-15	618	273	111	39	Nil	Nil

**Table 5:** The microorganism destruction activity of Cu-Ag/SBA-15 at 0.25 g level

Catalysts used	Bacterial count (CFU/ml) Samples at different dilutions					
	10^–1^	10^–2^	10^–3^	10^–4^	10^–5^	10^–6^
Raw water	9 × 10^8^	9.1 × 10^7^	8.9 × 10^6^	9.1 × 10^4^	9.1 × 10^3^	9.1 × 10^2^
0.25Cu-0.75Ag/SBA-15	Nil	Nil	Nil	Nil	Nil	Nil
0.5Cu-0.5Ag/SBA-15	109	19	Nil	Nil	Nil	Nil
0.75Cu-0.25Ag/SBA-15	214	118	28	17	Nil	Nil

**Table 6:** The microorganism destruction activity of Cu-Ag/SBA-15 at 0.5 g level

Catalysts used	Bacterial count (CFU/ml) Samples at different dilutions					
	10^–1^	10^–2^	10^–3^	10^–4^	10^–5^	10^–6^
Raw water	9 × 10^8^	9.1 × 10^7^	8.9 × 10^6^	9.1 × 10^4^	9.1 × 10^3^	9.1 × 10^2^
0.25Cu-0.75Ag/SBA-15	Nil	Nil	Nil	Nil	Nil	Nil
0.5Cu-0.5Ag/SBA-15	11	Nil	Nil	Nil	Nil	Nil
0.75Cu-0.25Ag/SBA-15	26	Nil	Nil	Nil	Nil	Nil

In summary, a variety of Cu-Ag/SBA-15 nanoalloy catalysts were synthesized using the incipient wetness impregnation method, and their ability to suppress microorganisms in drinking water was evaluated. Among the series of catalysts prepared, 0.25Cu-0.75Ag/SBA-15 catalyst exhibits exceptional activity in controlling the microorganisms. The exceptional activity of 0.25Cu-0.75Ag /SBA-15 catalyst over other Cu-Ag/SBA-15 catalysts can be attributed to its large surface area compared with other catalysts.

## Data Availability

Data is provided within the manuscript.
